# It’s Just the Flu...or Is It?

**Published:** 2018-01-01

**Authors:** Pamela Hallquist Viale

I received my flu shot at the end of September, right when my provider’s office received their shipment of vaccine. Avoidance of the flu is high on my list, so I am always astonished when I meet friends or neighbors who avoid the vaccine because of fear of getting "sick" from the shot or simply because they don’t think they’ll get the flu. It’s a mystery to me. Although this year’s vaccine is stated to have about 10% effectiveness against current strains of flu (it is always a guessing game when deciding which strains are going to be most problematic each year), the flu vaccine can contribute to shorter periods of sickness and decreased symptoms, even if you do get the flu.

## CALIFORNIA’S FLU EPIDEMIC

In California, where I live, the numbers of influenza patients are rising precipitously. Pharmacies have run out of common flu medicines, and the death toll is frighteningly high. In fact, it was reported last week that 27 people younger than 65 (it’s much more difficult to get numbers on patients older than 65, so this number is undoubtedly higher) have died of the flu since October (Karlamangla, 2018). To demonstrate how significant this is, at this time last year, the death toll was three! Emergency rooms are packed with influenza patients, and larger numbers of older patients are showing up with the most dangerous of combinations—flu plus pneumonia—which can be fatal. And of course, our primary patient population is always considered at higher risk, considering the disease and immune suppression that occurs frequently with cancer.

It was probably not the best of times for me to begin reading *Pale Rider: The Spanish Flu of 1918 and How It Changed the World* by Laura Spinney. My guess is that people who avoid the flu vaccine are those that may take the disease less seriously or underestimate the dangers of a flu epidemic. Reading this excellent history makes for a terrific, albeit frightening, read, but it also reminded me of several important facts about the Spanish flu.

## THE SPANISH FLU

The Spanish flu is considered one of the worst human disasters of all time. The disease infected a third of the people on Earth, killing approximately 50 to 100 million people, or 2.5% to 5% of the existing population (Spinney, 2018). More people may have died of the Spanish flu than in both the first and second world wars combined. It was also responsible for the largest number of dead since the Black Death (referring to the bubonic plague of the Middle Ages). And the pandemic permanently changed significant aspects of our society, including political, social, and family structures. The Spanish flu struck right when populations were migrating quickly and far from home, and when soldiers were sent to the first world war to fight. The infection spread fast and furiously; strangely, the group most at risk for death included those aged 20 to 40 (vs. the very young and elderly). People died very quickly, some within hours of displaying symptoms—and those symptoms were horrific. Most patients simply drowned in their own secretions, as their lungs hemorrhaged and blood-tinged froth appeared from their noses and mouths (Billings, 2005). Some patients displayed mahogany spots on their cheekbones, a harbinger of the dreaded cyanosis that later appeared, until the patient’s skin would appear almost entirely black (Spinney, 2018).

The American Public Health Association (APHA) issued recommendations to prevent the spread of disease and infection. They correctly identified the method of disease transmission (spreading from nose and throat discharge from infected persons) and recognized that prevention of contagion by well-ventilated rooms and the use of disinfection and sterilization would be useful. More importantly, perhaps, as reported in an early issue of the *Journal of the American Medical Association*, the APHA also believed that the best way to prevent infection was through the use of vaccines, although study of influenza vaccination was not yet sophisticated enough in its development.

## INFLUENZA IN IMMUNE-COMPROMISED PATIENTS

Our current influenza outbreak is certainly not as dire as the Spanish influenza pandemic, but caution is always important. As viruses mutate and the world grows more interconnected, the possibility of a future pandemic exists. Therefore, I do pay attention to the need for influenza vaccination and the identification of the current and most problematic strains of influenza at the moment.

In our patient population, influenza may pose a vital threat (Hermann et al., 2017). Although specific data are hard to find, a European study noted that influenza in immune-compromised and cancer patients may produce high rates of pneumonia and mortality, and concluded that early and rapid diagnosis with prompt antiviral treatment is essential for best outcomes (Hermann et al., 2017). Therefore, as advanced practitioners caring for this population of patients, rigorous education of our patients and early identification of flu symptoms are critical. Patients with cancer should get a flu shot, NOT the nasal spray (nasal spray vaccines are weak live viruses and can be a risk for an immune-compromised patient; American Cancer Society, 2017).

As I write this in January 2018, it’s not too late to get your flu shot. The infection usually peaks in February, so if you haven’t been vaccinated, get to your provider and get your flu shot. Although the flu is usually several days of miserable symptoms for most, the prospect of more serious outcomes, including death, can occur. Influenza is a serious business for patients with cancer and for you and your family.

## References

[1] American Cancer Society. (2017). Should people with cancer get a flu shot?. https://www.cancer.org/treatment/treatments-and-side-effects/physical-side-effects/infections/should-i-get-a-flu-shot.html.

[2] Billings M (2005). The influenza pandemic of 1918.. https://virus.stanford.edu/uda/.

[3] Hermann B, Lehners N, Brodhun M, Boden K, Hochhaus A, Kochanek M, Meckel K, Mayer K, Rachow T, Rieger C, Schalk E, Weber T, Schmeier-Jürchott A, Schlattmann P, Teschner D, von Lilienfeld-Toal M (2017). Influenza virus infections in patients with malignancies -- characteristics and outcome of the season 2014/15. A survey conducted by the Infectious Diseases Working Party (AGIHO) of the German Society of Haematology and Medical Oncology (DGHO).. *European journal of clinical microbiology & infectious diseases : official publication of the European Society of Clinical Microbiology*.

[4] Karlamangla S (2018). Severe flu brings medicine shortages, packed ERs and a rising death toll in California.. http://www.latimes.com/local/california/la-me-ln-flu-surge-20180106-htmlstory.html.

[5] Spinney L (2018). *Pale rider: The Spanish flu of 1918 and how it changed the world.*.

